# DGKζ Plays Crucial Roles in the Proliferation and Tumorigenicity of Human glioblastoma

**DOI:** 10.7150/ijbs.35193

**Published:** 2019-07-20

**Authors:** Xuefeng Gu, Guoqing Wan, Nianhong Chen, Jinhong Li, Bing Chen, Yeling Tang, Wangxian Gu, Cuiting Jin, Jihong Meng, Peng Zhang, Li Liu, Zhifang Yang, Changlian Lu

**Affiliations:** 1 Collaborative Research Center, Shanghai University of Medicine & Health Sciences, Shanghai, PR China; 2 College of Fundamental Medicine, Shanghai University of Medicine & Health Sciences, Shanghai, PR China; 3 Department of Neurosurgery, Jiangmen Central Hospital, Jiangmen, Guangdong, PR China; 4 Department of Neurosurgery, Affiliated Hospital of Guangdong Medical University, Zhanjiang, Guangdong, PR China; 5 College of Clinical Medicine, Shanghai University of Medicine & Health Sciences, Shanghai, PR China; 6 Shanghai Key Laboratory of Molecular Imaging, Shanghai University of Medicine & Health Sciences, Shanghai, PR China

**Keywords:** Diacylglycerol kinase zeta (DGKζ), glioblastoma, phosphorylation, cell proliferation, tumorigenicity

## Abstract

Glioblastoma is one of the most malignant brain cancers in adults, and it is a fatal disease because of its untimely pathogenetic location detection, infiltrative growth, and unfavorable prognosis. Unfortunately, multimodal treatment with maximal safe resection, chemotherapy and radiation has not increased the survival rate of patients with glioblastoma. Gene- and molecular-targeted therapy is considered to be a promising anticancer strategy for glioblastoma. The identification of novel potential targets in glioblastoma is of high importance. In this study, we found that both the mRNA and protein levels of diacylglycerol kinase ζ (DGKζ) were significantly higher in glioblastoma tissues than in precancerous lesions. The silencing of DGKζ by lentivirus-delivered shRNA reduced glioblastoma cell proliferation and induced G0/G1 phase arrest. Moreover, knockdown of DGKζ expression in U251 cells markedly reduced *in vitro* colony formation and *in vivo* tumorigenic capability. Further study showed that DGKζ inhibition resulted in decreases in cyclin D1, p-AKT and p-mTOR. Moreover, the rescue or overexpression of DGKζ in glioblastoma cells demonstrated the oncogenic function of DGKζ. In conclusion, these studies suggest that the suppression of DGKζ may inhibit the tumor growth of glioblastoma cells with high DGKζ expression. Thus, DGKζ might be a potential therapeutic target in malignant glioblastoma.

## Introduction

Glioblastoma multiforme (GBM) is reported to be the most malignant brain tumor. It represents approximately 70% of glial tumors in the central nervous system of humans. Generally, the peak incidence of GBM occurs around 30-40 years of age 1. GBM is the second greatest cause of mortality in patients younger than 34 years of age. GBM is deadly due to its untimely pathogenetic location detection, infiltrative growth and high recurrence rate. Despite advanced improvements in surgery skill, chemotherapeutic drugs, and stereotaxic radiotherapy, the median overall survival time varies between 12 and 15 months 2. Therefore, it is very important to find new therapeutic methods to suppress GBM growth and progression.

The functional study of genes or proteins that influence the occurrence and development of GBM will help us understand the molecular mechanisms of the pathogenesis of this fatal disease and identify effective therapeutic methods. Lipids are a component of the cell membrane and play an essential role in many cellular events; lipid levels are regulated by lipid-metabolizing enzymes, such as phospholipases and lipid kinases 3. Diacylglycerol kinase (DGK) is a lipid kinase that catalyzes the phosphorylation of diacylglycerol (DAG) to produce phosphatidic acid (PA), which uses ATP as a phosphate donor 4-6. Both DAG and PA serve as second messengers in cell signaling. DAG regulates a variety of important signal transducers, including protein kinase C (PKC) 7 and RAS guanyl releasing protein (RasGRP) 8, 9, while PA plays a key role in regulating signaling proteins such as aPKC, c-Raf, chimaerin 10-12, mTOR 13, 14 and PIP5K 15. Thus, DGK acts as a molecular hub for many signaling pathways through regulating intracellular DAG and PA levels.

To date, ten DGK isozymes have been reported in mammals, including α, β, γ, δ, η, κ, ε, ζ, ι and θ. These isozymes are divided into five groups (I, II, III, IV and VI) according to their structural characteristics 16. They exhibit obviously different tissue distributions, expression patterns and biological functions. There is growing evidence that DGKs play an important role in human malignant tumors. For example, several studies showed aberrant expression of DGKα in hepatocellular carcinoma (HCC), mammary carcinoma, melanoma and GBM 17-19, and it is thought to serve as a potential target for drug design 19. The important crystal structure and properties of Ca^2+^-bound DGKα-EF were reported by Takahashi 3. Moreover, increased expression of DGKη was found in malignant lung carcinoma with *EGFR* or *KRAS* mutations, and silencing DGKη suppressed tumor cell growth 20. DGKγ plays a tumor suppressor role in HCC and colon cancer by inhibiting cell proliferation and cell migration 21, 22. It should be noted that diacylglycerol kinases ζ (DGKζ) is characterized as type IV due to its myristoylated alanine-rich C-kinase substrate (MARCKS), ankyrin, and PDZ binding domains 23, 24. Similar to other DGKs, DGKζ is also reported to be abnormally expressed in human colorectal cancer cells, and it is indispensable for the proliferation of cancer cells. In addition, DGKζ knockout attenuated the invasiveness of MDA-MB-231 mammary carcinoma and PC-3 prostate tumor cells 25. Interestingly, Topham et al. 7 found that the MARCKS domain of DGKζ could regulate the nuclear localization of DGKζ. This finding suggests that DGKζ might play different roles in different cellular events. Torres-Ayuso P et al. 13 reported that DGKζ regulates mTORC1 and lipometabolism in human colorectal tumor cells through SREBP-1. Moreover, Tanaka T et al. 26 found that DGKζ is involved in regulating the crosstalk between NF-kB and p53, two major signaling pathways in cell survival and death. Collectively, these findings suggest that regulation of DGKζ expression might be an effective way to inhibit the proliferation of metastatic tumor cells, including glioblastoma cells. It will be very important to explore this possibility as there are no studies of DGKζ expression patterns and potential roles in patients with glioblastoma.

In this work, we have presented evidence that DGKζ mRNA and protein levels were significantly higher in tumor tissues from glioblastoma patients than in precancerous lesions. Silencing DGKζ by lentivirus-delivered shRNA reduced glioblastoma cell proliferation and induced G0/G1 phase arrest. The rescue and overexpression of DGKζ in glioblastoma cells demonstrated the function of DGKζ as an oncogene. Moreover, knockdown of DGKζ expression in U251 cells markedly reduced colony formation, endothelial cell tube formation *in vitro* and tumorigenic capability* in vivo*. Furthermore, western blots showed that the possible downstream molecules of DGKζ were phosphorylated mTOR and AKT. In conclusion, these studies suggest that the suppression of DGKζ may inhibit tumor development in glioblastoma cells with high DGKζ expression. DGKζ appears to be a potential therapeutic target in malignant glioblastoma.

## Materials & Methods

### Tissue Samples

Human glioblastoma tissues and corresponding para-carcinoma tissues were obtained from patients who were diagnosed at the Department of Neurosurgery, Affiliated Hospital of Guangdong Medical University (Zhanjiang, China), from 2014 to 2016. The pathological-grade tumors were confirmed according to the WHO 2000 criteria. Normal brain specimens were from victims of automobile accidents without tumors. All patients enrolled in this work gave written informed consent. The protocol was approved by the Ethics Committee of Guangdong Medical University. The clinical samples were washed with normal saline and then immediately frozen in liquid nitrogen for RNA extraction or preserved in 10% formalin for immunohistochemical staining.

### Immunohistochemistry

Immunohistochemical staining for DGKζ was performed on clinical specimens as previously described 27. The slices were stained with a rabbit polyclonal antibody against human DGKζ purchased from LSbio (Seattle WA 98121, USA). Immunoperoxidase staining was performed using an ABC Peroxidase Standard Staining Kit (Pierce, Rockford, IL, USA) according to the manufacturer's instructions.

### Cell Culture

U87 MG, HS683, SHG-44, U373, LN229, and U251 cells were obtained from the American Type Culture Collection (ATCC, Manassas, VA, USA). H4 and HEB cells were purchased from Bena Culture Collection (Kunshan, China). According to the supplier's recommendations, all cells were cultured in Dulbecco's modified Eagle's medium (DMEM, Gibco BRL, MD, USA) supplemented with 10% FBS, 100 μg/ml streptomycin, and 100 U/ml penicillin at 37°C in a humidified incubator containing 5% CO_2_.

### DGKζ silencing and overexpression

We created lentiviral constructs that inducibly express DGKζ-targeting shRNAs (GeneBank Accession: NM_003646) or control shRNA. The shRNA sequences were as follows: DGKζ-shRNA1, 5'-TACTCTGAAAGCAAGCAAGAA-3'; DGKζ-shRNA2, 5'- AGTGGTGTGTGATGGAATGGACT-3'; and scrambled, 5'-TTCTCCGAACGTGTCACGT-3'. Lentiviruses were packaged and purified to yield Lv-DGKζ-shRNA1, Lv-DGKζ-shRNA2, and Lv-scr-shRNA, which were used to infect glioblastoma cells. DGKζ expression after targeted shRNA lentivirus infection was examined by qRT-PCR or western blot analysis on the fourth day.

DGKζ overexpression (DGKζ OE) and negative control lentiviral vectors were obtained from GeneChem Co., Ltd. (Shanghai, China). After infecting U251 cells for 4 days, the expression of DGKζ was examined by western blot analysis.

### RNA extraction and qRT-PCR

TRIzol reagent (Invitrogen, Karlsruhe, Germany) was used for total RNA isolation. Reverse transcription PCR was performed using a one-step RNA PCR kit (TaKaRa, China). SYBR-Green Master PCR Mix (Applied Biosystems, Life Technologies, USA) was used for qRT-PCR. All qRT-PCR primer pairs were purchased from SABiosciences. Data collection was performed using an ABI PRISM 7500 sequence detection system (Applied Biosystems, Life Technologies, USA). β-actin was selected as endogenous control to eliminate errors in reverse transcription PCR and to standardize the measurement of RNA expression levels. The relative quantification value for each target gene was compared with the control (β-actin), which was expressed as 2^-(Ctest-Ccontrol)^ (Ctest and Ccontrol are the mean cycle threshold (Ct) value differences after normalization to β-actin). The relative expression levels of the samples were determined by semi-log plot.

### Western blot analysis

Total protein was extracted from glioblastoma cells using RIPA buffer (Thermo Fisher Scientific, Waltham, MA). The protein concentrations were examined using a BCA Protein Quantification Kit (Yeasen, Shanghai, China). Samples containing 30 μg protein were subjected to SDS-PAGE and electrophoretically transferred to nitrocellulose membranes (Millipore, Bedford, MA, USA). The membranes were blocked with solution containing 5% nonfat dry milk and then incubated overnight at 4 °C with specific primary antibodies, which included DGKζ (LS-C101153), anti-cyclin D1, anti-mTOR, anti-phospho-mTOR (Ser2448), anti-AKT and anti-phospho-AKT(Ser473) (1:1000, CST, Beverly, MA) antibodies, followed by incubation with a secondary antibody (goat anti-rabbit IgG (HRP) from Abcam, ab6721). After the membranes were washed, the blots were developed using an ECL kit (Yeasen, Shanghai, China).

### Edu assay

Infected cells were cultured in 96-well plates and incubated with 50 mmol/L EdU (RiboBio) for 2 hours. Cells were fixed with 4% paraformaldehyde for 30 minutes at room temperature. After permeabilization with 0.5% Triton-X-100, the cells were incubated with fluorescent staining solution for 30 minutes. Then, the DNA in the cells was stained with Hoechst 33342 for 30 minutes. Cells were then observed with a confocal fluorescence microscope (Zeiss, Thornwood, NY).

### Flow cytometry analysis of the cell cycle

U251 cells were cultured in 6-well plates and treated for 48 hours. Subsequently, the transfected cells were collected after washing twice with PBS and then fixed in 70% ice-cold ethanol at -20 °C overnight. The next day, the fixed cells were stained with 50 μg/mL PI (propidium iodide, ABCONE, Shanghai, China) containing 50 μg/mL RNase A (DNase-free) for 15 minutes at room temperature. Flow cytometric analysis was performed using a flow cytometer (FACSCalibur, Becton Dickinson) and FlowJo software (Tree Star).

### Colony formation assay

For the colony formation assay, U251 and U87 MG cells (500 per well) were seeded in a 6-well plate at 24 h post-transfection and cultured in medium containing 10% FBS for 10 days. When the colonies reached more than 50 cells, they were fixed with 10% formaldehyde for 15 minutes and stained with an 8% crystal violet solution for 10 minutes; then, the colonies were imaged using a digital camera. Each experiment was repeated three times.

### Tube formation assay

A 96-well plate was coated with growth factor-reduced Matrigel (BD Biosciences, USA); then, 2 × 10^5^ HUVECs/ml in complete medium and 100 μl of U251 medium were added to each well. One hundred microliters of 60% diluted tumor conditioned medium from cells treated with DGKζ shRNA1 or shRNA2 was added to each well, and tube formation was monitored with an inverted microscope after 6 hours of incubation. The total tube length in each well was measured and calculated using ImageJ. Experiments were performed in triplicate.

### Tumorigenesis assay

Female nude athymic mice (7 weeks old, weighing approximately 18 g) were purchased from Shanghai Sippr BK Laboratory Animals Ltd. (Shanghai, China). The mice were maintained in a specific pathogen-free (SPF) animal facility. All animals received humane care according to the National Research Council's guidelines. U251 cells infected with Scr-shRNA or DGKζ-shRNA1 lentivirus were suspended in PBS at a final concentration of 5 × 10^7^ cells/ml. A 100-μl aliquot of the cell suspension (approximately 5 × 10^6^ U251 cells) was injected into the axilla region of nude mice in the treatment and control groups. The mice were sacrificed 28 days post-injection, and tumor growth was examined. The tumor sizes and volumes were determined through external measurements and calculated by the formula: V = [L×W^2^] × 0.5 (V =volume, L = length and W=width). The data were analyzed using Student's t-test, and differences were considered significant at *P* < 0.05.

### Statistical methods

GraphPad Prism 6.0 software (GraphPad Software, CA, USA) was used for data analysis. The data are expressed as the mean±standard deviation (SD). Student's t-test or one-way analysis of variance (ANOVA) followed by Dunnett's post hoc test was utilized to analyze the differences between the two groups. *P* < 0.05 was considered statistically significant.

## Results

### DGKζ expression was upregulated in glioblastoma

**Figure 1:**
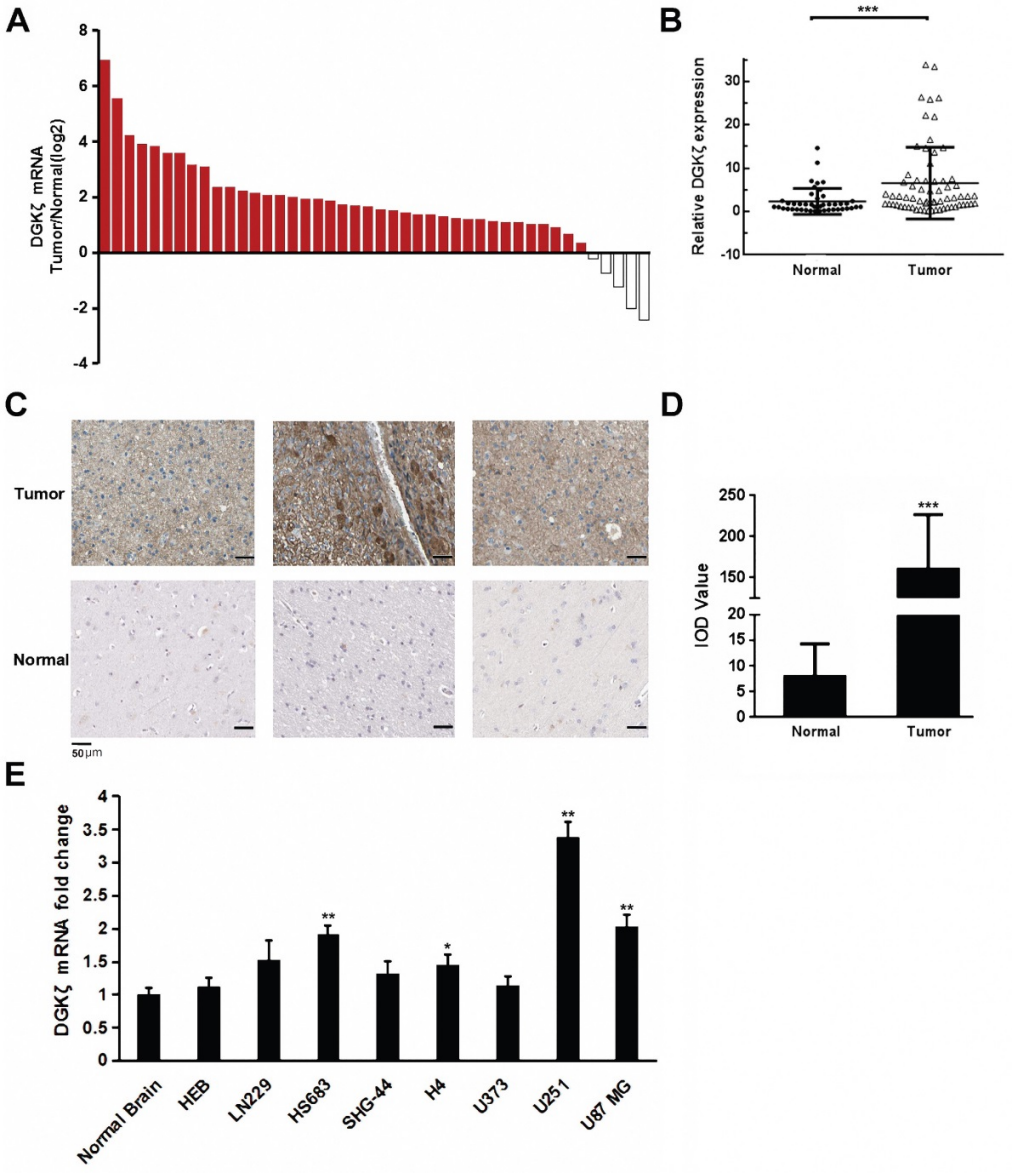
** DGKζ is highly expressed in glioma cells**. (A, B) DGKζ mRNA expression was obviously higher in glioma tissues than in paired normal tissues in most patients (39/44). (C, D) Representative immunohistochemical staining for DGKζ expression in human glioma tissues (upper) and precancerous tissues (lower). The average IOD value was obtained by analyzing five fields per slide with Image-Pro Plus software (v. 6.0) and recorded in histograms. (E) DGKζ mRNA levels in normal brain tissues and glioma cell lines detected by qRT-PCR analysis. The results represent at least three separate experiments. Error bars: ± S.D. * *P* < 0.05, ** *P* < 0.01, *** *P* < 0.001.

DGKζ mRNA expression was first examined in glioma and corresponding para-carcinoma tissues from 44 clinical patients diagnosed with gliomas. The qRT-PCR results showed that DGKζ mRNA expression was obviously higher in glioma tissues than in paired normal tissues from most (39 of 44) patients (Fig. 1A and B). Subsequently, immunohistochemical staining was performed to confirm the protein expression of DGKζ in the glioma tissues. DGKζ levels appeared to be higher in malignant glioma tissues than in precancerous tissues from glioma patients (Fig. 1C and D). However, there was no significant correlation between DGKζ and glioma tumor grades because of the limited sample size (Table 1). To further detect the correlation between DGKζ expression and glioma, we used eight cell lines, HEB, LN229, HS683, SHG-44, H4, U373, U251 and U87 MG, to examine DGKζ mRNA expression by qRT-PCR analyses. The qRT-PCR results showed that DGKζ mRNA levels were higher in 4 cell lines than in normal brain tissue samples; U251 and U87 MG cells had the highest expression levels (Fig. 1E). In conclusion, our results suggested that DGKζ expression was upregulated in gliomas.

### Overexpression of DGKζ in U373 and H4 glioma cells enhanced cell proliferation

**Figure 2:**
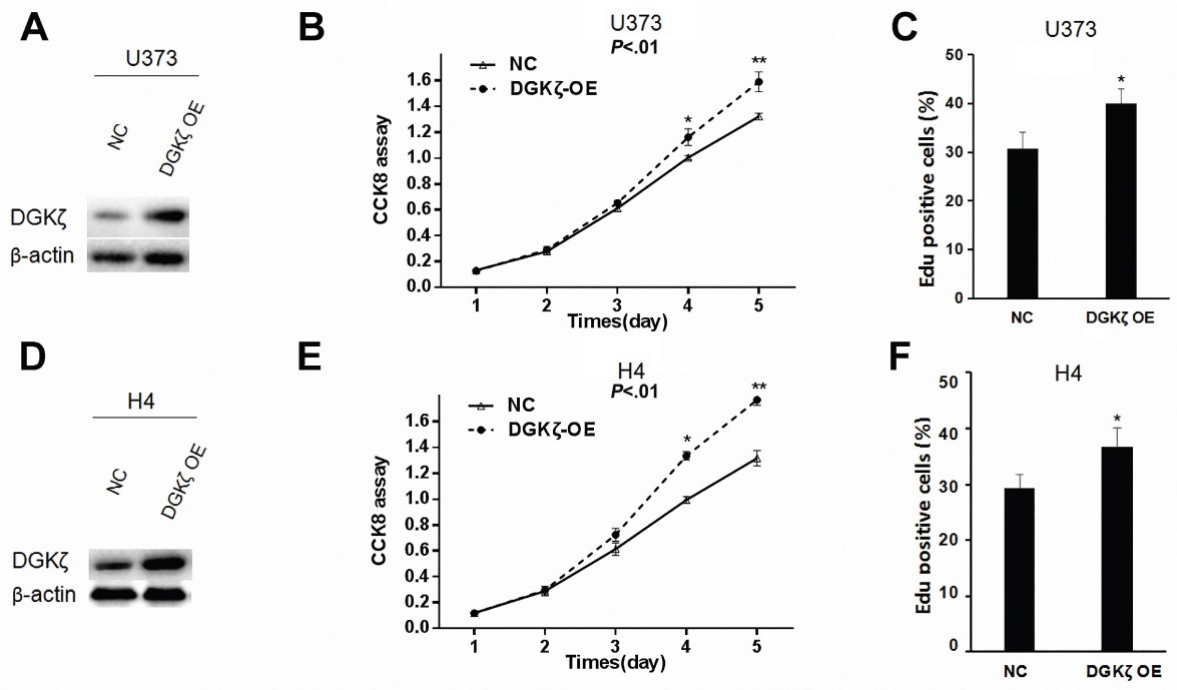
** DGKζ promotes glioma cell proliferation *in vitro*.** (A) U373 cells were infected with lentivirus carrying DGKζ (DGKζ OE) or negative control (NC). The cell lysates were immunoblotted with a DGKζ antibody, and β-actin was used as a loading control. (B) *In vitro* growth of U373-NC and U373-DGKζ-OE cells was measured using CCK8 assays. (C) *In vitro* growth of U373-NC and U373-DGKζ-OE cells was measured using EdU assays. (D) H4 cells were infected with lentivirus carrying DGKζ (DGKζ OE) or negative control (NC). The cell lysates were immunoblotted with a DGKζ antibody, and actin was used as a loading control. (E) *In vitro* growth of H4-NC and H4-DGKζ-OE cells was measured using CCK8 assays. (F) *In vitro* growth of H4-NC and H4-DGKζ-OE cells was measured using EdU assays. Error bars: ± S.D. * *P* < 0.05, ** P < 0.01.

Gain- or loss-of-function studies were performed to investigate the potential role of DGKζ in glioma cells. First, we overexpressed DGKζ in U373 and H4 glioma cells with low endogenous DGKζ expression (Fig. 1C, 2A and 2D). The effect of DGKζ on glioma cell proliferation was measured using the CCK8 assay. A significant increase in the cell growth curve after transfection with DGKζ was found in both U373 and H4 cells (Fig. 2B and E). This result indicated that cell proliferation was enhanced due to the upregulation of DGKζ. To further confirm this result, we carried out EdU proliferation assays. Consistent with the CCK8 analysis results, both U373 and H4 cells showed higher EdU-positive staining after overexpression of DGKζ (Fig. 2C and F). These results suggest that DGK plays a role in promoting glioma cell proliferation.

### Knockdown of DGKζ in U251 and U87 MG glioma cells inhibited cell proliferation, while rescue of DGKζ increased cell proliferation

**Figure 3:**
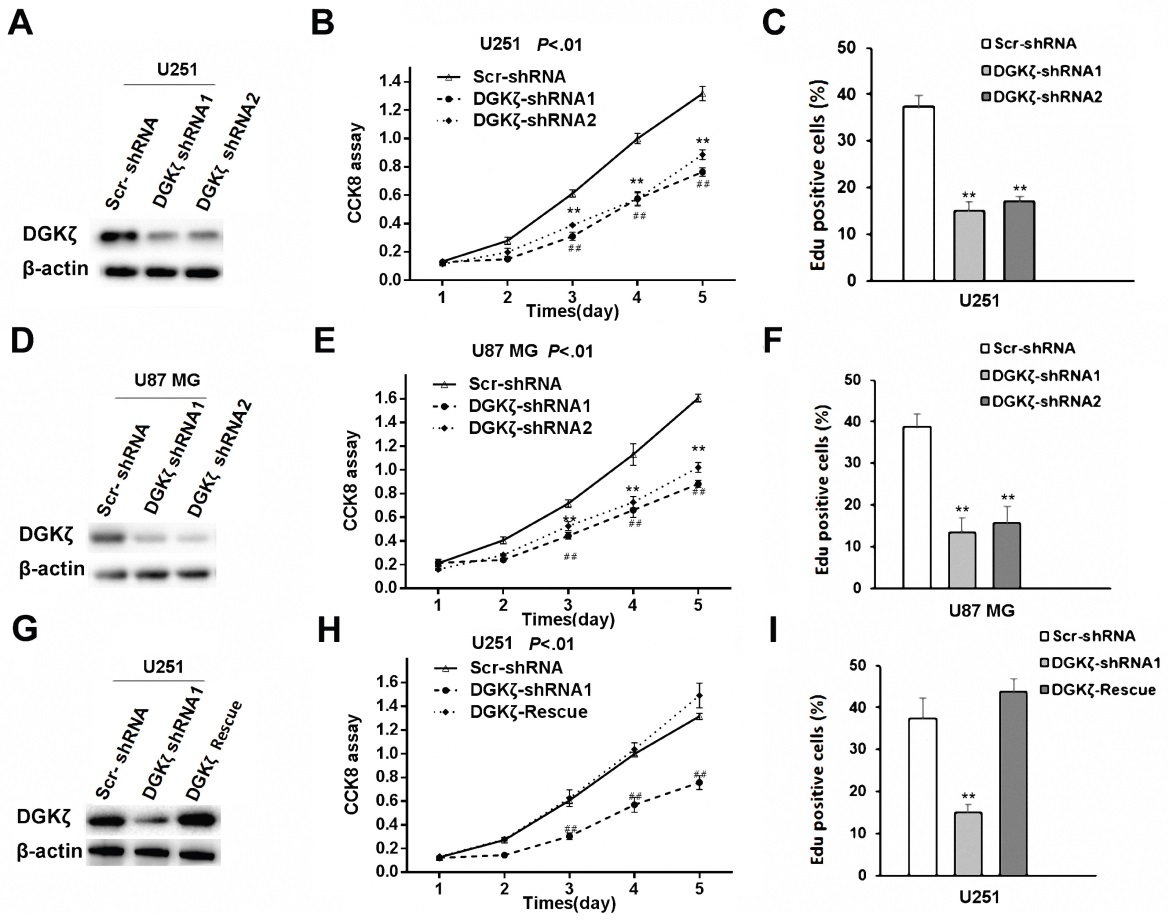
** Inhibition of DGKζ remarkably suppresses cell viability in glioma cells with high DGKζ expression**. (A) U-251 cells were transfected with DGKζ shRNA1 or DGKζ shRNA2 and scrambled shRNA as a control (Scr shRNA). DGKζ levels were detected by immunostaining with a DGKζ antibody, and actin was used as a loading control. (B) *In vitro* growth of U-251/Scr shRNA and U-251/ DGKζ shRNA1 or shRNA2 cells was measured using CCK8 assays. (C) *In vitro* growth of U-251/Scr shRNA and U-251/ DGKζ shRNA cells was measured using EdU assays. (D) U87 MG cells were transfected with DGKζ shRNA1 or DGKζ shRNA2 and scrambled shRNA as a control (Scr shRNA). DGKζ levels were detected by immunostaining with a DGKζ antibody, and actin was used as a loading control. (E) *In vitro* growth of U87 MG/Scr shRNA and U87 MG/DGKζ shRNA1 or shRNA2 cells was measured using CCK8 assays. (F) *In vitro* growth of U87 MG Scr shRNA and U87 MG/DGKζ shRNA cells was measured using EdU assays. (G) DGKζ protein expression in U251 cells infected with a vector expressing DGKζ after DGKζ-shRNA1-mediated DGKζ knockdown. (H) Cell viability curves of the three U251 cell groups over 5 days were evaluated by CCK8 assay. (I) *In vitro* growth of U251 Scr shRNA, U251/DGKζ shRNA1 and U251-DGKζ-rescue cells was measured using EdU assays. Error bars: ± S.D. * *P* < 0.05, ** P < 0.01.

Subsequently, high DGKζ expression in the U87 MG and U251 cell lines was knocked down (Fig. 3A and D). Five days after DGKζ-shRNA knockdown in the U87 MG and U251 cell lines, CCK8 analysis was performed, and the results showed cell growth inhibition compared with that in Scr-shRNA-infected cells. The viability of DGKζ-shRNA-infected cells was obviously lower (*P* < 0.01) than that of Scr-shRNA-infected control cells (Fig. 3B and E).

An EdU proliferation assay was employed as an alternative method to measure cell proliferation. Consistent with the CCK8 analysis results, both U251 and U87 MG cells showed lower EdU-positive staining levels after transfection with DGKζ shRNA than after transfection with control shRNA (Fig. 3C and F).

A rescue experiment was used to confirm the specificity of the effects of DGKζ knockdown in glioma cells. DGKζ expression was enhanced after transfecting the DGKζ vector in DGKζ-shRNA1-infected U251 cells (Fig. 3G). Furthermore, enhancing DGKζ expression rescued the decrease in cell viability induced by RNAi-mediated DGKζ depletion (Fig. 3H and I).

### Inhibition of DGKζ significantly augments the tumor growth of DGKζ-expressing glioma cells

**Figure 4:**
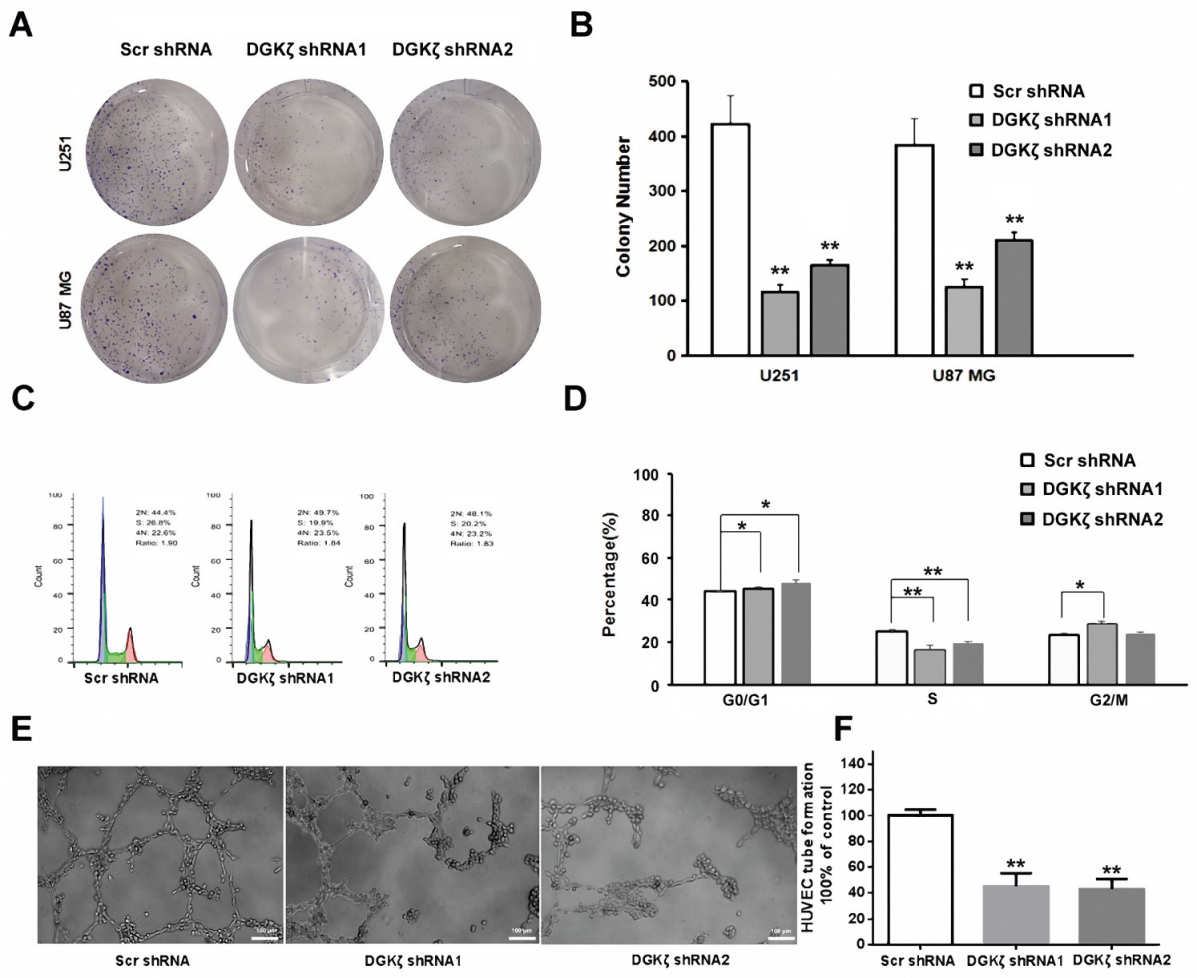
** Inhibition of DGKζ prominently suppresses tumor development in glioma cells.** (A, B) Colony formation assay of cells from the three groups. Cells were seeded at 500 cells/well and allowed to form colonies for 10 days. The colonies were stained with crystal violet and observed. (C, D) Cell cycle analysis of cells from the three groups as determined by PI staining and FACS analysis (left). The percentages of cells in each phase represent the mean ± S.D. of three independent experiments (right). * *P* < 0.05, ** *P* < 0.01. (E, F) Representative images from the HUVEC tube-formation assay. Quantified data correspond to the mean ± S.D. of three independent experiments (right). ** *P* < 0.01.

To further investigate the effects of DGKζ inhibition in glioma cells, colony formation, flow cytometry analysis of the cell cycle, and tube formation assays were performed *in vitro.* Transfection of U251 and U87 MG cells with DGKζ shRNA resulted in a significant decrease in the cell numbers of each colony compared with nonsense shRNA transfection. The number of colonies with more than 50% cells was also decreased in DGKζ shRNA-transfected cells (Fig. 4A and B).

After U251 cell PI staining, flow cytometry analysis showed that DGKζ inhibition led to a significant increase in G0/G1 phase arrest but a decrease in the number of S phase cells (Fig. 4C and D). These data demonstrated that DGKζ inhibition results in U251 cells in the quiescent phase.

Moreover, a tube formation assay revealed that knockdown of DGKζ could inhibit the formation of extravascular blood vessels (Fig. 4E and F).

**Figure 5:**
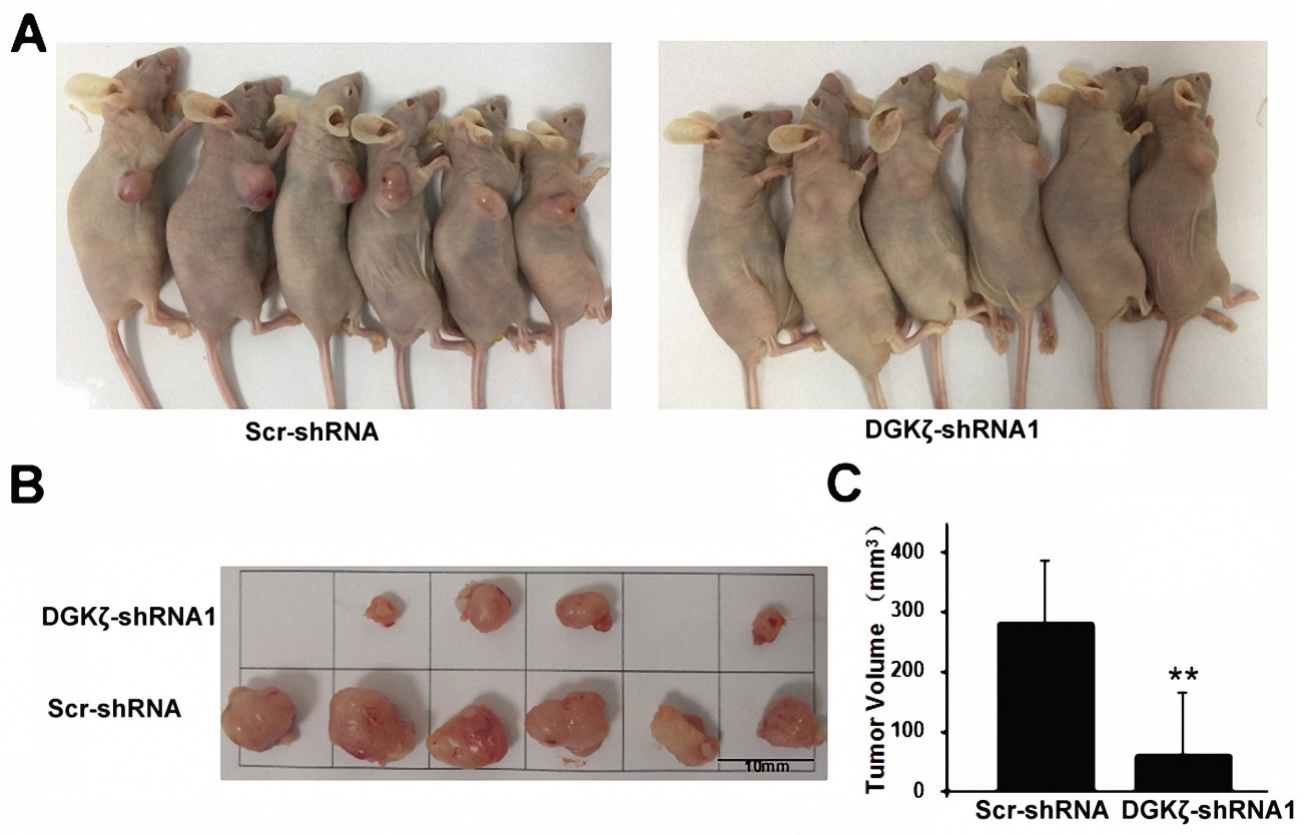
** DGKζ inhibits glioma cell proliferation *in vivo*.** (A) Scr-shRNA and DGKζ-shRNA groups of nude mice were injected in the axilla region with the corresponding infected U251 cells (10^6^ cells per mouse). After 4 weeks, the mice were sacrificed, and their tumors were examined (n=6) (B). (C) Quantified data correspond to the mean ± S.D. of two groups (n=6). ** *P* < 0.01.

We next examined the *in vivo* effect of DGKζ using a subcutaneous xenograft nude mouse model. Significant inhibition of tumor growth relative to the control group (82.8%; *P* < 0.01) was recorded in the DGKζ knockdown group (Fig. 5). Considering all the experiments together, the most significant result obtained was that the inhibition of DGKζ expression suppressed the tumor growth of glioma cells with high DGKζ expression.

### DGKζ silencing caused the downregulation of cyclin D1, AKT phosphorylation and mTOR phosphorylation

**Figure 6:**
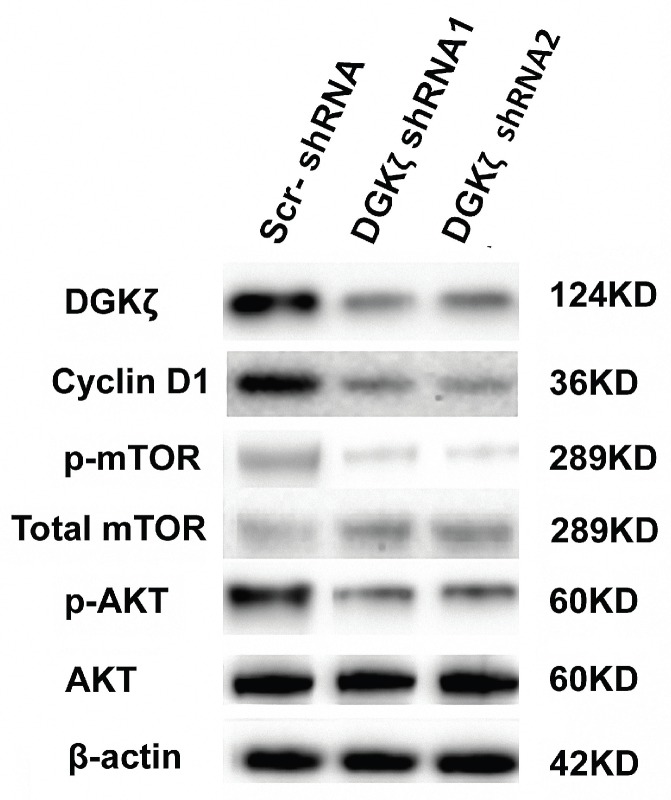
** DGKζ silencing decreases cyclin D1, phosphorylated AKT and phosphorylated mTOR levels in U251 cells.** U251 cells were transfected with lentiviruses expressing the shRNAs. DGKζ, cyclin D1, phosphorylated mTOR and phosphorylated AKT protein levels were assayed by western blots. Western blot analyses indicated that DGKζ silencing decreased cyclin D1 and phosphorylated mTOR and phosphorylated AKT protein levels. One representative result is shown.

We next investigated the mechanism by which DGKζ inhibition suppressed glioma growth *in vitro* and *in vivo*. Because the role of DGKζ in carcinogenesis as well as in tumor progression has been poorly documented, only a few studies have reported that DGKζ has an anti-apoptotic function that is dependent on the mTOR pathway 28-31. Moreover, the flow cytometry results showed that U251 glioma cells were arrested at the G0/G1 phase. PI3K/AKT/mTOR is an important intracellular signal transduction pathway involved in the cell cycle; thus, AKT/mTOR-associated signaling pathways were examined first. Western blot analysis showed that the phosphorylation levels of mTOR and AKT were lower in DGKζ-shRNA-infected cells than in control cells (Fig. 6), while there was no evident change in total AKT and mTOR, indicating that DGKζ silencing inactivated the AKT/mTOR pathway in glioma cells through a phosphorylation/dephosphorylation mechanism. We also found the downregulation of cyclin D1 in DGKζ knockdown cells. The main function of cyclin D1 is to promote cell proliferation and G1/S phase transition by binding and activating cyclin-dependent kinase CDK4, phosphorylating cyclin inhibitor protein (Rb) in the G1 phase, dissociating the phosphorylated Rb protein from its binding E2F transcription factor, and initiating the transcription of genes in the cell cycle. The molecular mechanisms of how these genes work downstream of DGKζ require future evaluation.

## Discussion

Gliomas can occur at any age, regardless of sex and race. Based on their histopathology, gliomas can be subdivided into grades I‑IV according to the World Health Organization (WHO) classification for CNS tumors 32. Grade III and IV gliomas are more aggressive and difficult to cure due to the frequent dysfunction of tumor suppressors and oncogenes. Despite the combination of surgery, radiotherapy, and chemotherapy, the prognosis for patients remains very poor, and less than 5% of patients with malignant glioma survive 5 years 33. Presently, the only chemotherapeutic drug is temozolomide; Everolimus is a molecule-targeted drug for subependymal giant cell astrocytoma (SEGA) associated with tuberous sclerosis (TS) that cannot be treated surgically, and Avastin is a monoclonal antibody (mAb) drug that is the only second-line treatment for malignant glioma approved by the FDA 34. As we better understand the molecular mechanisms underlying glioma development, molecular targeted therapy has been proposed as a promising treatment for glioma. For instance, BRAF 35, EGFR 36, EZH2 37, MET 38 and mTOR 39 have been reported as potential protein targets for glioma treatment. However, because of the complexity of the occurrence and development of glioma, none of these targets can completely cure glioma; thus, more novel therapeutic targets are urgently needed.

In the present study, we demonstrated that DGKζ mRNA and protein levels were significantly higher in tumor tissues from glioblastoma patients than in precancerous lesions. DGKζ knockdown by lentivirus-delivered shRNA reduced glioblastoma cell proliferation and induced G0/G1 phase arrest. In addition, the rescue or overexpression of DGKζ in glioblastoma cells further validated the function of DGKζ as an oncogene. Moreover, knockdown of DGKζ expression in U251 cells markedly reduced colony formation, endothelial cell tube formation* in vitro* and tumorigenic capability* in vivo*. At the molecular level, western blots showed that the possible downstream molecules of DGKζ were phosphorylated mTOR and AKT. In conclusion, these studies suggest that the suppression of DGKζ could inhibit the tumor development of glioblastoma cells with high DGKζ expression, thus highlighting DGKζ as a potential therapeutic target in malignant glioblastoma.

The underlying mechanism of DGKζ inhibition of glioma cell growth was explored in this work. DAG and PA are important membrane components and biosynthetic precursors of phospholipids. DAG conversion into PA by DGK is the first step of replenishing PIP2 in the so-called “PIP2 cycle”, a reaction that provides an important lipid substrate, not only for the subsequent hydrolysis by phospholipase C (PLC) but also for the production of PIP3 by the action of PI3 kinase (PI3K). This information indicated that DGKζ might play important roles in the signaling pathways related to PIP2 or PI3K. Moreover, the PI3K/AKT/mTOR pathway has been regarded as an important signaling pathway that regulates many intracellular upstream signaling pathways that influence cell growth, metabolism, survival polarity, angiogenesis, and vesicle trafficking. In our study, AKT/mTOR was markedly inhibited by DKGζ knockdown in U251 cells, indicating the interesting role of DKGζ in glioma. Moreover, our results demonstrated that DGKζ inhibition arrested the cell cycle at the G0/G1 phases. The AKT/GSK3β pathway induces cell cycle progression by enhancing cyclin D1 expression. The reason for this finding might be that these cells were screened during the process of tumor development, and some compensatory DGKζ-independent pathway was already activated in these screened cells to support cell survival and proliferation. However, the exact mechanisms require further investigation.

## Figures and Tables

**Table 1:** Associations between DGKζ expression and the clinicopathological characteristics of 44 glioma patients

Clinicopathological characteristics		n	< 0	0-1	1-2	> 2	*P* value
Age	≤ 60	36	3	2	16	15	0.303
	> 60	8	2	1	4	1	
Sex	male	26	4	2	11	9	0.761
	female	18	1	1	9	7	
Grade	I-II	15	3	2	5	5	0.295
	III-IV	29	2	1	15	11	

*P* values were detected by the chi-square test.

## Abbreviations

DGKζ: diacylglycerol kinase zeta
shRNA: short hairpin RNA
DMEM: Dulbecco's modified Eagle's medium
FBS: fetal bovine serum
Scr-shRNA: scrambled shRNA
qRT-PCR: quantitative real-time PCR
CCK8: Cell Counting Kit 8
PI: propidium iodide
PIP2 cycle: phosphatidylinositol-4,5-bisphosphatecycle
PI3K: phosphatidylinositol 3-kinase
AKT: V-akt murine thymoma viral oncogene homolog
mTOR: mammalian target of rapamycin
